# Genetic diversity pattern reveals the primary determinant of burcucumber (*Sicyos angulatus* L.) invasion in Korea

**DOI:** 10.3389/fpls.2022.997521

**Published:** 2022-11-15

**Authors:** Soo-Rang Lee, Dong Chan Son

**Affiliations:** ^1^ Department of Biology Education, College of Education, Chosun University, Gwangju, South Korea; ^2^ Division of Forest Biodiversity and Herbarium, Korea National Arboretum, Pocheon, South Korea

**Keywords:** invasive species, multiple introductions, landscape genetics, population structure, range expansion, *Sicyos angulatus* L.

## Abstract

Biological invasion is a complex process associated with propagule pressure, dispersal ability, environmental constraints, and human interventions, which leave genetic signatures. The population genetics of an invasive species thus provides invaluable insights into the patterns of invasion. Burcucumber, one of the most detrimental weeds for soybean production in US, has recently colonized Korea and rapidly spread posing a great threat to the natural ecosystem. We aim to infer the determinants of the rapid burcucumber invasion by examining the genetic diversity, demography, and spread pattern with advanced genomic tools. We employed 2,696 genome-wide single-nucleotide polymorphisms to assess the level of diversity and the spatial pattern associated with the landscape factors and to infer the demographic changes of 24 populations (364 genotypes) across four major river basins with the east coastal streams in South Korea. Through the approximate Bayesian computation, we inferred the likely invasion scenario of burcucumber in Korea. The landscape genetics approach adopting the circuit theory and MaxEnt model was applied to determine the landscape contributors. Our data suggested that most populations have experienced population bottlenecks, which led to lowered within-population genetic diversity and inflated population divergences. Burcucumber colonization in Korea has strongly been affected by demographic bottlenecks and multiple introductions, whereas environmental factors were not the primary determinant of the invasion. Our work highlighted the significance of preventing secondary introductions, particularly for aggressive weedy plants such as the burcucumber.

## Introduction

Biological invasion leaves traceable genetic tracks on each stage of invasion, which offer indispensable information for making proper management plans and effective control practices (Arredondo et al., 2018; Shirsekar et al., 2021). Colonization of a species takes place in a complex manner associated with four consecutive stages: an initial introduction with a small-sized population, colonization followed by dramatic population growth, and population expansion (Richardson et al., 2000; Pysek et al., 2004; Theoharides and Dukes, 2007; Blackburn et al., 2011). Although the primary ecological and evolutionary forces driving each invasion process may greatly differ (Theoharides and Dukes, 2007; Reed et al., 2020), genetic changes are inevitable due to the demographic changes and the accompanying altered selective pressure. Accordingly, invasion processes can be reconstructed by trailing genetic marks through a population genetics study, which is particularly important for invasive species with lack of well-documented records.

In the initial stages, introduced species likely experience both genetic and stochastic challenges because species colonization is often associated with low propagule pressure, i.e., the combined effect of the propagule size and number (Baker, 1955; Lande, 1988; Simberloff, 2009; Blackburn et al., 2015; Dlugosch et al., 2015; Blackburn et al., 2020). The small founding populations derived from the limited propagule pressure accompany several genetic challenges, e.g., deprivation of genetic diversity, loss of advantageous alleles, elevated genetic load, and altered selection pressure (Frankham, 1995; Blackburn et al., 2015; Dlugosch et al., 2015). However, despite the complications, some introduced species successfully colonize and widely spread in the new area. The genetic paradox (Allendorf and Lundquist, 2003) can partly be explained by the continued propagule pressure (Simberloff, 2009; Blackburn et al., 2020). The colonizing alien species might continuously receive propagules from genetically divergent sources, ameliorating the problems caused by the small founding populations. The flow of continued propagule pressure could increase the existing genetic variation, and it could also bring up new genotypes that are better adapted to novel environments (Novak and Mack, 1993; Mccauley, 1997; Sax et al., 2007; Dlugosch et al., 2015; Barker et al., 2019). In fact, multiple introductions of divergent lineages and/or congeneric species are rather commonly observed in widespread invasive plants, e.g., saltcedar, *Tamarix* spp., and Japanese knotweed, *Reynoutria japonica* (Lee, 2016; VanWallendael et al., 2021).

During the last phase of biological invasion, several factors including population characteristics, e.g., growth rates and landscape heterogeneity, affect the rates and direction of the spread (Theoharides and Dukes, 2007). Environmental gradients across the landscape, dispersal vectors, and demographic constraints are the main determinants of the range expansion, which largely influence the genetic architecture of the invasive species (von der Lippe and Kowarik, 2007; Hulme, 2009; Wilson et al., 2009; Sherpa and Després, 2021). Several studies showed the importance of dispersal vectors, e.g., water courses and roads, as potential corridors (Christen and Matlack, 2009; Aronson et al., 2017; Lee et al., 2018; Ward et al., 2020). Also, several studies tested the barrier effects of environmental factors such as temperature and precipitation (Koncki and Aronson, 2015; Lee et al., 2018). In plants, among-population genetic variability is strongly influenced by barriers and corridors of dispersal. Landscape genetics, the amalgamation of molecular population genetics and landscape ecology (Manel et al., 2003), grants a powerful tool for understanding the dynamics of range expansion, particularly for invasive plants. It can provide ecological and evolutionary insights into the landscape factors controlling the dispersal of plants (Ouborg et al., 1999). Anthropogenic aspects should also be taken into consideration because potential effects of strong and complex human interventions such as multiple introductions and human-mediated long-distance dispersal may rescind the landscape effects.

Burcucumber, *Sicyos angulatus* L. (Cucurbitaceae; 2n = 24), is an annual vine widely distributed in the east side of North America (Walker, 1973; Waminal and Kim, 2015). The plant commonly occupies open spaces and moist areas such as river banks, flood plains, agricultural land, fences along roads, and rarely woods (Walker, 1973). In the last few decades, burcucumber has become one of the most troublesome invasive plants in the northern hemisphere (Smeda and Weller, 2001; Kurokawa et al., 2009; Lee et al., 2015). According to the Global Biodiversity Information Facility (GBIF), the species occurs from over 4,000 localities in 10 countries, of which eight countries are out of the native range. Given the violent vining habit over small native plants and crops, the plant poses enormous economic—particularly for corn and soybean production—and ecological threats (Walker, 1973; Smeda and Weller, 2001; Farooq et al., 2017). In one growing season, about 18,000 g of biomass can be expected from a single burcucumber plant (Esbenshade et al., 2001). The adult plants easily outgrow the natives replacing the natural vegetation, which poses great threats to biodiversity and ecosystem integrity.

Burcucumber exhibits a series of life history traits, contributing to be a notorious invasive plant. The species is primarily an outcrossing plant (monoecious, maybe selfing—see European and Mediterranean Plant Protection Organization, 2010) flowering from summer to fall with three to 15 fertile flowers on each capitate head (Arifin and Okamoto, 2020). Diverse groups of generalist insects including bees and flies pollinate burcucumber, which have been proposed as a key component of invasion success for outcrossing plants (Ward and Johnson, 2013; Montesinos et al., 2016; Arifin and Okamoto, 2020). The plant primarily reproduces with seeds, and the seeds’ annual production is fairly high (Esbenshade et al., 2001; Farooq et al., 2017; Arifin and Okamoto, 2020). The seeds germinate throughout the whole growing season and grow fast reaching 30 cm tall in a day under favorable moist and temperature conditions (Walker, 1973; Smeda and Weller, 2001). When fully mature, the fruits are covered by spines that can be attached to passing animals efficiently and assist dispersal to rather distant areas (Walker, 1973). Burcucumber also has an ability to colonize a wide range of habitats due to its physiological plasticity. For example, the plant can germinate and grow in areas with 5°C–40°C temperatures and survive from wet to semiarid soil moisture conditions. It can even tolerate mildly saline soil (Farooq et al., 2017).

In Korea, there is no well-documented information on the introduction time and the invasion pathways of burcucumber. However, based on the scattered records, it is presumed that the species was transferred from North America as a grafting stock for cucumbers and watermelons (National Institute of Environmental Research, 2005). The first observation of burcucumber was made by a local citizen in 1989, but there was a complete lack of supporting information such as a proper publication and/or herbarium sheets (National Institute of Environmental Research, 2005). The first herbarium record of burcucumber was found in 1990, and it was collected from a central region (National Institute of Environmental Research, 2005; Korea National Arboretum, 2019). In Japan, one of the neighboring countries of Korea, the first occurrence of burcucumber was reported much earlier than the one in Korea (in 1950; Kurokawa et al., 2009). Given the frequent commercial trades and travels between the two neighboring countries, the introduction date of burcucumber in Korea was probably before the 1990s. Over the past few decades, burcucumber has rapidly expanded its range throughout South Korea replacing native plants (National Institute of Environmental Research, 2005; Moon et al., 2007). The plant is currently one of the 10 most abundant invasive plants in Korea, particularly in the riparian system (Park and Lee, 2018). On average, ca. 40% of the riparian vegetation is composed of burcucumber (Park and Lee, 2018). Unfortunately, the causal mechanisms of the successful burcucumber invasion in Korea have never been explored, thus remaining elusive.

The aims of our study are twofold: 1) to assess the genetic diversity pattern that might have been associated with multiple introductions and the demographic history and 2) to determine the contributing landscape factors during the rapid range expansion. Former genetic studies of burcucumber in Japan suggested the possibility of multiple introductions, but the sample size and the number of molecular markers used were limited (Kurokawa et al., 2009; Kobayashi et al., 2012). Despite the aggressive infestations in many places across the globe, the determinants of the successful burcucumber invasion have not been empirically explored. We employed a large number of molecular markers sampled from the whole genome and collected many populations throughout the entire country to provide a detailed and precise assessment of population structure. We also inferred the invasion history by competing likely invasion scenarios with the approximate Bayesian computation (ABC) approach. Additionally, through a species distribution model (SDM) and correlation analyses, we developed the isolation-by-resistance (IBR) model and compared it with the conventional isolation-by-distance (IBD) model to identify the contributing landscape factors during the rapid range expansion of burcucumber invasion in Korea.

## Materials and methods

### Sample collection

**Figure 1:**
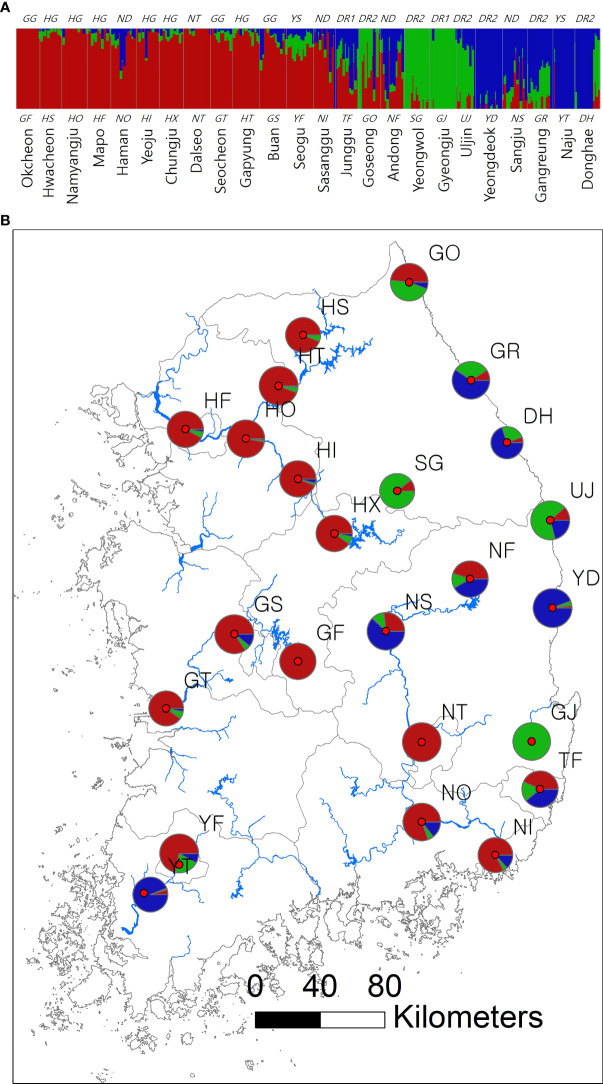
Spatial structure of 24 *Sicyos angulatus* populations throughout South Korea. **(A)** The bar chart of the clustering assignment for *K* = 3 assessed from fastSTRUCTURE based on 2,696 SNP loci sampled across 346 burcucumber genotypes. The vertical white lines separate each population from one another. Colors indicate locus assignment into each of the three clustering groups. See **Table 1** for population abbreviations. **(B)** Map of burcucumber collection sites in South Korea. Pie charts summarized the clustering assignments of individual genotypes within each population. The pie sizes reflect the relative sample size of each population.

During the summer of 2021, we collected young burcucumber leaves of 383 individuals from 24 populations across four river basins (Han, Geum, Nakdon, Youngsan) and rivers along east coast regions in South Korea (**Table 1**; **Figure 1**). We sampled populations at ~50-km intervals to identify broad-scale patterns of spatial divergence. An adult burcucumber stretches out the vines up to 7 m (Smeda and Weller, 2001). Thus, to avoid collecting multiple samples from a single plant, we carefully selected samples by distancing at least 30 m between the sampled plants. Leaf tissues were preserved at room temperature in a sealed plastic Ziplock bag with silica gel desiccant until further use. We isolated the genomic DNA with the dried leaf tissues using DNeasy Plant Mini Kit (Qiagen, Hilden, Germany) following the manufacturer’s protocol. To check the quality, the extracted DNAs were run on a 1% agarose gel through gel electrophoresis. We measured the quantity of the DNAs using a Qubit 4 Fluorometer (Thermo Fisher Scientific, MA) and stored them at −20°C.

**Table 1:** Locality information of burcucumber collection sites in South Korea.

Location	Abbreviation	Region (river basin)	Assigned group	N	Lon	Lat	He [ ± sd]	Na [ ± sd]	Na_Rare
Gwangju Seogu	YF	YS1	pop1	16	126.83178	35.14983	0.18 [0.004]	1.52 [0.011]	1.54
Jeonnam Naju	YT	YS1	pop2	13	126.63753	34.98481	0.07 [0.002]	1.34 [0.009]	1.28
Chungbuk Okcheon	GF	GG1	pop1	14	127.64600	36.28170	0.10 [0.003]	1.35 [0.009]	1.32
Chungbuk Gongju	GS	GG1	pop1	16	126.28494	35.61262	0.22 [0.003]	1.76 [0.009]	1.68
Chungbuk Seocheon	GT	GG1	pop1	13	126.74051	36.02049	0.22 [0.004]	1.63 [0.011]	1.72
Seoul Mapo	HF	HG1	pop1	14	126.86983	37.57463	0.24 [0.003]	1.78 [0.009]	1.73
Gangwon Hwacheon	HS	HG1	pop1	13	127.69903	38.09631	0.19 [0.004]	1.57 [0.010]	1.53
Gyunggi Gapyung	HT	HG1	pop1	16	127.52375	37.81537	0.20 [0.004]	1.67 [0.010]	1.58
Gyunggi Namyangju	HO	HG1	pop1	15	127.29314	37.52126	0.20 [0.003]	1.69 [0.009]	1.57
Gyunggi Yeoju	HI	HG1	pop1	14	127.65445	37.29693	0.23 [0.004]	1.76 [0.008]	1.66
Chungbuk Chungju	HX	HG1	pop1	14	127.90429	36.99143	0.18 [0.004]	1.47 [0.012]	1.72
Gyungbuk Andong	NF	ND1	na	14	128.84023	36.72724	0.21 [0.004]	1.57 [0.011]	1.62
Gyungbuk Sangju	NS	ND1	pop2	15	128.25370	36.44429	0.23 [0.004]	1.70 [0.009]	1.64
Daegu Dalseo	NT	ND1	pop1	16	128.48928	35.82371	0.20 [0.004]	1.61 [0.010]	1.53
Gyungnam Haman	NO	ND1	pop1	15	128.48172	35.38012	0.21 [0.004]	1.66 [0.009]	1.58
Busan Sasanggu	NI	ND1	pop1	13	128.97720	35.18888	0.24 [0.003]	1.81 [0.008]	1.7
Ulsan Junggu	TF	DR1	na	14	129.28935	35.54980	0.22 [0.004]	1.61 [0.010]	1.65
Gangwon Yeongwol	SG	DR2	pop3	15	129.24042	35.81558	0.04 [0.001]	1.36 [0.009]	1.29
Gangwon Goseong	GO	DR2	na	13	128.34508	37.22291	0.09 [0.003]	1.31 [0.010]	1.21
Gangwon Gangreung	GR	DR2	pop2	15	128.45221	38.38352	0.16 [0.004]	1.51 [0.010]	1.32
Gangwon Donghae	DH	DR2	pop2	15	128.87427	37.83201	0.17 [0.004]	1.46 [0.010]	1.47
Gangwon Uljin	UJ	DR2	pop3	11	129.11578	37.48184	0.06 [0.002]	1.34 [0.009]	1.48
Gangwon Yeongdeok	YD	DR2	pop2	16	129.40468	37.04363	0.17 [0.003]	1.63 [0.010]	1.24
Gyungbuk Gyeongju	GJ	DR1	pop3	16	129.40585	36.55596	0.08 [0.003]	1.33 [0.010]	1.59

The abbreviation represents the population abbreviations. N, sample size; Lat and Lon, geographic coordinates in decimal degrees. The assigned group indicates the cluster of each local population assigned for the ABC computation. He, expected heterozygosity; Na, number of alleles; Na_Rare, number of alleles adjusted by the population sample sizes; YS, Youngsangang watershed; GG, Geumgang watershed; HG, Hangang watershed; ND, Nakdonggang watershed; DR, East coast streams. ± sd in the parenthesis is the standard deviation.

### Restriction site-associated DNA library preparation and genotyping

3RAD (Bayona-Vásquez et al., 2019) was employed for genotyping. The method is a reduced representation approach modified from the well-known ddRADseq (Peterson et al., 2012) by adding a third restriction enzyme to cut adapter dimers to increase the adapter ligation efficiency, which likely improves the amplified read yield. We prepared the 3RAD library as follows. We first digested the genomic DNAs using *Eco*RI-HF and *Xba*I. *Nhe*I was added for dimer cleaving (all enzymes from Thermo Fisher Scientific). We then ligated the index adapters (BadDNA, University of Georgia, Athens, GA) in a 15-µl reaction mixture containing 100 ng DNA, 8 µl master mix, and 1 µl of each 5-µM adapter. We made sure the total volume of the master mix to be 15 µl containing 0.5 µl of each restriction enzyme and 1.5 µl of 10× FastDigest Buffer. The digestion and ligation were conducted simultaneously in a tube. The resultant products were incubated in a thermal cycler for 15 min at 37°C. We prepared 5 µl of the ligation mixture as follows: 0.5 µl of 10× Ligase Buffer, 100 units of T4 DNA ligase, 1.5 µl of 10 mM ATP, and 2 µl ultrapure water. The ligation mixture was added to each sample and incubated at 22°C for 20 min and 37°C for 10 min. We repeated the process twice then incubated the mixture at 80°C for another 20 min. The resultant products of the ligation were then examined by PCR amplification using Bioneer Multiplex PreMix. For the PCR, we prepared a 20-µl reaction volume containing 1 µl of iTru5 and iTru7 primers and 1 µl of adaptor-ligated DNA fragments. For the amplification, we used the following thermal cycler profile: 95°C for 10 min; 35 cycles of 95°C for 30 s, 60°C for 1 min, 72°C for 30 s; and 72°C for 5 min. After the ligation examination, we pooled 5 µl of the adaptor-ligated fragments from each sample in a 1.5-ml tube and purified the pooled samples using a 1:1.8 mixture of AmPure XP magnetic beads (Beckman Coulter, CA). Seventy percent of EtOH was used to wash the prepared samples, and the samples were left at room temperature for 15 min to air-dry. The dried samples were resuspended with 60 µl of TE buffer. We then eluted the purified DNA fragments from the magnetic beads. Amplification was carried out to enrich the adapter-ligated DNA fragments. For the amplification, a 50-µl reaction volume was prepared to contain 10 µl of the pooled DNA fragments, 5 µl of 5 µM iTru5 primer, 5 µl of 5 µM iTru7 primer, 10 µl of 5× HF Buffer, 1.5 µl of 10 µM dNTP, and 1 unit of Phusion DNA Polymerase. The amplification profile was as follows: 98°C for 2 min; 7 cycles of 98°C for 20 s, 60°C for 15 s, 72°C for 30 s; and 72°C for 5 min. Additional sample cleanup was conducted by using a 1:1.8 mixture of AmPure beads. Before the size selection, we washed the samples twice with 70% EtOH and resuspended the samples in 60 µl TE buffer. Following the manufacturer’s protocol, we selected the targeted size fragments (500-bp fragments ± 10%) using Pippin Prep (Sage Science, MA). We performed a final amplification with a 50-µl reaction mixture containing 5 µl of size-selected DNA, 3 µl of 5 µM P5 primer, 3 µl of 5 µM P7 primer, 1.5 µl of 10 µM dNTP, 10 µl of 5× HF Buffer, and 1 unit of Phusion DNA Polymerase. The thermal cycler profile was as follows: 98°C for 2 min, 98°C for 20 s, 61°C for 15 s, and 72°C for 45 s for 12 cycles; and 72°C for 5 min. The third cleanup (final) for the amplicons was administered with a 1:1.8 mixture of AmPure beads. We washed the amplicons twice with 70% EtOH and resuspended them in 35 µl ultrapure water. The complete 3RAD library was evaluated for quality and quantity through Agilent 2100 Bioanalyzer (Agilent Technologies, CA). The fully prepared library was run on an Illumina HiSeq X-10 platform using 2 × 150 paired-end sequencing at Macrogen Inc., Korea.

We demultiplexed, trimmed, and processed raw sequence data in Stacks ver. 2.41 (Rochette et al., 2019). To evaluate the quality of the raw reads, we applied the Phred score with the threshold of 10 using the process_radtag function. Given the lack of a fully assembled and annotated reference genome, the restriction site-associated DNA (RAD) loci were assembled *de novo*. For catalog assembly, we set the parameters as −m 3 and −M 3 in the ustacks function allowing a maximum of one mismatch between sample loci (−n 1, cstacks function; Paris et al., 2017). With the catalogs constructed, we called SNPs on a population function implemented in Stacks. We only extracted SNP loci if the loci were present in at least 80% of the samples within each population and shared by at least 12 populations (−p 12 and −r 0.8). To ensure the independence of SNP loci avoiding linkage disequilibrium (LD), we selected only the first SNP per locus (-write-single-snp). We further screened the SNP loci that significantly departed from the Hardy–Weinberg equilibrium (HWE, threshold = P < 10e-6; Li, 2011; Hess et al., 2012) to avoid the loci with extreme heterozygosity and assembly errors in Plink ver. 1.9 (Purcell et al., 2007). Finally, the genotypes with more than 30% missing calls and SNP loci with a minor allele frequency of ≤0.05 were pruned from the data set in Tassel 5.0 (Glaubitz et al., 2014).

### Data analysis

For genetic diversity computation, expected heterozygosity (*He*), observed heterozygosity (*Ho*), and the number of alleles (*Na*) were calculated in GenAlEx 6.5 (Peakall and Smouse, 2012). Given the varying number of individuals sampled across 24 populations, *Na* was adjusted using rarefaction curves to account for unequal sizes (**Table 1**; (Kalinowski, 2004) in HP-Rare (Kalinowski, 2005). Population divergence was estimated as pairwise F*_ST_* among 24 populations in Arlequin version 3.5 with 1,000 permutations for evaluating the statistical robustness (Excoffier and Lischer, 2010).

We tested the presence of recent bottlenecks for the 24 local populations with 261 unlinked SNPs subsampled from the total 2,696 SNPs (r^2^ < 0.1). The number of SNPs used for the population bottleneck test was reduced to ease the computational challenges. The population at mutation-drift equilibrium approximately shows an equal probability of a heterozygosity excess or a heterozygosity deficit. Populations that experienced a recent bottleneck would show widespread heterozygote excess on a significant number of loci. Excess of heterozygosity was assessed following Cornuet and Luikart (1996) in the software Bottleneck (Piry et al., 1999). We ran Bottleneck with the infinite allele model (IAM) and the stepwise mutation model (SMM). To estimate statistical robustness, we used the sign and Wilcoxon’s signed-rank test implemented in BOTTLENECK ver. 1.2.02 (Piry et al., 1999). We also tested if there is a significant mode shift from the equilibrium state in allele frequencies.

We examined genomic cluster assignment patterns by two approaches, principal coordinate analysis (PCoA) and Bayesian model-based assignment test. PCoA was carried out on Nei’s genetic distance computed from all 346 genotypes in GenAlEx. We inferred the number of randomly mating subgroups (*K*) using fastSTRUCTURE, an efficient alternative of the software STRUCTURE for large-size data such as genome-wide SNPs (Pritchard et al., 2000; Raj et al., 2014). For the prior, we chose “logistic,” which is more flexible with respect to population size variation and population structure (Raj et al., 2014). We ran a series of analyses with increasing *K* from 1 to 12 and repeated the process 20 times for cross-validation. The optimal *K* was then determined by the function “chooseK” in fastSTRUCTURE. The expected admixture proportion inferred was visualized using Pophelper, the R package (Francis, 2017; R Core Team, 2021).

### Inferring demographic history

We employed the approximate Bayesian computation (ABC) approach to further infer the demographic history of burcucumber invasion in Korea using DIYABC Random Forest (DIYABC-RF) version 1.0 (Collin et al., 2021). For the ABC computation, we subsampled 526 unlinked SNPs (r < 0.1) from the 2,696-final-SNP set to ease the computational challenges and secure the independence among markers. As the ABC approach assumes a non-continuous gene flow between populations, we delimited the populations to three clusters (populations 1 to 3) based on the result of clustering analysis, STRUCTURE, and the geographic features of the populations (see assigned groups in **Table 1** for the cluster information). The three populations (NF, TF, and GO) that are severely admixed were purged from the analysis to meet the assumption of the ABC computation. Given the lack of genotype data from the native region, we set up three unsampled “ghost populations” (populations 4 to 6). The ghost populations accounted for the unknown genetic sources that might have been introduced to Korea from the native area (Cornuet et al., 2010).

**Figure 2:**
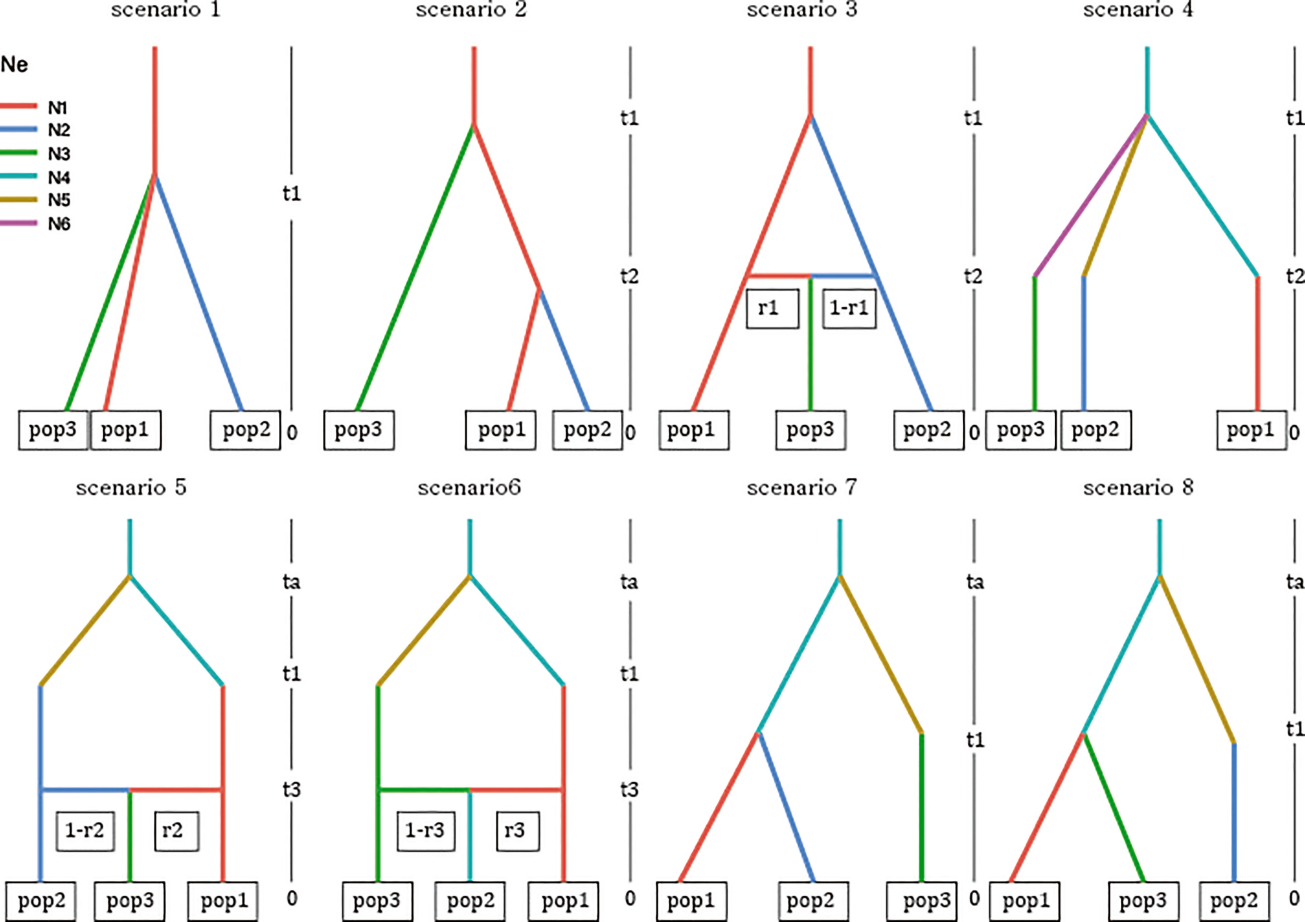
Graphical illustration of the eight scenarios of invasion history for burcucumber invasion in Korea competed on the approximate Bayesian computation analysis implemented in DIYABC-RF v. 1.0. See **Table 1** for cluster information used in the analysis (pop1–pop3). Acronyms: ta, time of divergence in the origin; t1, time of introduction; t2 and t3, time of divergence or admixture in the introduced region (Korea); r1–r3, recombination rates; N1–N3, effective population sizes of sampled populations; N4–N6, effective population sizes of unsampled populations (ghost populations) from the origin.

Based on the clustering results, the current distribution, and prior information on burcucumber invasion in Korea, we constructed eight demographic scenarios of burcucumber invasion (**Figure 2**). For scenarios 1–3, we assumed a single-source introduction from the native region with (scenario 2) or without subsequent divergence (scenario 2) and an admixture event (scenario 3). We hypothesized multiple introductions of genetically divergent sources from the native region for scenarios 4–8. In the scenarios, initially, a population in the origin has diverged at the time of ta (divergence in the native area might have dated back to glacial divergence 1,000 < ta < 10,000) then the diverged sources introduced into Korea at t1 (relatively recent, possibly 31 < t1 < 100). We set up the time range based on the occurrence records and the generation time (1 year for the annual vine such as burcucumber).

For checking the level of errors on the scenario choice and parameter estimation accuracy, we checked the location of an observed data set over the prior data space using a PCA plot depicting the simulated data set retained from the training and observed data following Collin et al. (2021). We generated 4,000 training data sets for each of the eight tested scenarios and set up the prior distribution of parameters as uniform. The priors for the effective population sizes of the sampled populations and the unsampled ghost populations were set to 10–1,000 and 100–10,000, respectively. The parameters of divergence times were set in an ascending order (t1 > t2 > t3, between 10 and 100) except for ta, i.e., the divergence time in the origin (101 < ta < 10,000). We selected the best scenario and estimated the posterior probability for the choice of the scenario in a random forest algorithm implemented in DIYABC-RF with 500 trees generated for each analysis (Collin et al., 2021). To estimate the parameters incorporated in the selected scenario (scenario 7), we used training data of 30,000 simulated data and set the number of trees as 1,000.

### Landscape genomic analysis

The hypothesis “a given landscape gradients affects the dispersal of an organism” can be investigated by calculating the correlation between genetic similarity and distance and resistance along the gradients (Manel et al., 2003; Manel and Holderegger, 2013). Following McRae and Beier (2007), we compared the conventional IBD (isolation by distance) and IBR (isolation by resistance) to determine how landscape factors shape the population structure and affect the connectivity among the burcucumber populations in South Korea. The most widely used Mantel tests do not consider the non-independence of the pairwise distances (Yang, 2004; Guillot and Rousset, 2013; Manel and Holderegger, 2013). Therefore, we employed a maximum-likelihood population effect (MLPE) method that uses a linear mixed effect model for IBD and IBR estimations (Clarke et al., 2002; van Strien et al., 2012; Phillipsen et al., 2015). In the MLPE model, the fixed effects were geographic and resistance distances, whereas the random effect was the individual population effect. The MLPE models were fit by the residual maximum likelihood (REML) approach using R package ‘lme4’ (Bates et al., 2014; R Core Team, 2021). The marginal r^2^, an approximation for the fixed effects in a mixed effect model (Nakagawa and Schielzeth, 2013), was also computed as a measure of a goodness of fit in the r package MUMIN ver. 1.42.1 (Barton, 2009). For the genetic distance metric, we used both F*_ST_* and Slatkin’s linearized F*_ST_* [F*_ST_*/(1 − F*_ST_*)] as proposed by Rousset (1997). We then compared the model fit between the F*_ST_* and the linearized F*_ST_* by the marginal r^2^. F*_ST_* outperformed all distance models over the linearized F*_ST_* (see **Table S1**); therefore, we finalized our landscape genetics models to three models derived from F*_ST_* with the three different distance measures (Euclidean distance, log-transformed Euclidean distance, and resistance distance). The best model was selected using the conditional AIC (cAIC, adjusted AIC for the mixed effect model; Greven and Kneib, 2010) in R 4.1.0 package “CAIC4” (Saefken and Ruegamer, 2014; R Core Team, 2021).

In the IBD models, it is more appropriate to use log-transformed Euclidean distances than simple Euclidean distances when dealing with a species with two-dimensional habitat types (Rousset, 1997). The distribution pattern appears to be rather complex. In the collected populations for the present study, the habitat types are largely composed of two. The populations from the east coastal streams and a few populations within the four river basins are the two-dimensional habitat types, whereas most populations within the four river basins (about half of collected populations) are linear. We, therefore, calculated both simple and log-transformed Euclidean distances as predictors for the genetic distances between the 24 sampled populations.

For resistance distance computation, we employed a species distribution model (SDM) to determine the habitat suitability of burcucumber. We assumed that the low habitat suitability likely impeded the dispersal between population pairs; thus, an SDM can be applied to assess resistance distances. The maximum entropy method (MaxEnt ver. 3.4.4; Phillips et al., 2006) was used to develop an SDM for burcucumber in South Korea. The MaxEnt approach is one of the most commonly used algorithms for developing SDMs and performs more effectively than the other methods known when only presence data are available (Elith et al., 2006; Jo et al., 2018). We combined our field survey with the occurrence data retained from the third National Natural Environment Survey (2006–2013), specimen information from the Korean National Arboretum (http://www.nature.go.kr/main/Main.do) and Naturing (https://www.naturing.net/, observation data archiving site collected from citizen scientists), which generated 879 presence data points. We used 19 climate variables (downloaded from www.worldclim.org; **Table S1**) and a digital elevation model (DEM) of South Korea (downloaded from https://egis.me.go.kr) to construct the current SDM. We clipped all other data sets with DEM using the “Extract by Mask” function of ArcGIS10.6.1 (ESRI) and adjusted the geographic dimensions of the data used in the analysis to the South Korea DEM by a 5-m resolution. All parameters of MaxEnt were set to default with 50 replicates and 5,000 maximum iterations. We selected the best-fitting model using the area under the receiver operating characteristic curve (AUC), which corresponds with the perfect classification model around a value of 1.0. The produced value of SDM was inverted by the “Reclassify” function of ArcGIS for resistance raster. We finally computed the pairwise resistance distances in Circuitscape ver. 4.0 (McRae, 2006). For data conversion, we employed the software Circuitscape for the ArcGIS toolbox. With the inverted SDM as a raster resistance map, we produced a current map and a voltage map using Circuitscape. We used pairwise modeling on each focal site with an eight-neighboring-connection scheme on a raster resistance map. To calculate the length of resistance distances, we used the “Cost Path” function of ArcGIS.

## Results

The sequenced 3RAD library was 211 Gbp and produced ~14 million reads with an average GC content of 39.5%. We called over 150,000 SNP loci from the initial SNP identification. After a series of screening processes, 2,696 SNPs remained for the downstream analyses. On average, the genetic diversity of burcucumber varied across populations although the differences were not significant (**Table 1**). The adjusted allele numbers ranged from 1.21 (GO; see **Table 1** for the population acronyms) to 1.73 (HF; **Table 1**). We found the lowest mean *He* (= 0.06) in the UJ population at which the number of collected samples was the smallest, whereas the highest mean *He* was observed in HF (mean *He* = 0.24; **Table 1**). Pairwise genetic divergence among populations estimated as F*_ST_* greatly varied across the population pairs (0.10, NI/NO – 0.77, SG/UJ; **Table 2**). The mean F*_ST_* was 0.37, and all F*_ST_* values were statistically robust (*P* < 0.01). Based on the sign and Wilcoxon’s rank tests, most populations sampled in our research experienced recent population bottlenecks (**Table 3**). In five populations (YT, GF, GJ, DH, and YD), the P values were high (*P* > 0.05) showing that the populations are likely in the equilibrium state of population demography (**Table 3**). However, among the five populations, three populations showed a significant allele frequency mode shift compared with the equilibrium state, indicating the possibility of recent population bottlenecks (**Table 3**).

**Table 2:** Mean pairwise F*_ST_* values computed from 2,696 SNPs among 24 *Sicyos angulatus* populations in South Korea.

	YF	YT	GF	GS	GT	HF	HS	HT	HO	HI	HX	NF	NS	NT	NO	NI	TF	GJ	SG	GO	GR	DH	UJ	YD
YF	0.00																							
YT	0.48	0.00																						
GF	0.44	0.63	0.00																					
GS	0.27	0.43	0.35	0.00																				
GT	0.29	0.47	0.34	0.18	0.00																			
HF	0.23	0.41	0.29	0.16	0.17	0.00																		
HS	0.29	0.49	0.44	0.25	0.28	0.19	0.00																	
HT	0.29	0.48	0.43	0.24	0.28	0.19	0.10	0.00																
HO	0.27	0.46	0.40	0.23	0.26	0.15	0.16	0.16	0.00															
HI	0.25	0.43	0.22	0.16	0.18	0.09	0.22	0.22	0.17	0.00														
HX	0.39	0.56	0.50	0.33	0.36	0.25	0.37	0.37	0.31	0.27	0.00													
NF	0.32	0.44	0.44	0.25	0.29	0.23	0.30	0.29	0.27	0.24	0.35	0.00												
NS	0.27	0.34	0.40	0.21	0.25	0.19	0.25	0.25	0.23	0.20	0.35	0.20	0.00											
NT	0.28	0.48	0.38	0.24	0.25	0.19	0.27	0.27	0.23	0.18	0.38	0.29	0.23	0.00										
NO	0.27	0.42	0.31	0.20	0.21	0.16	0.25	0.25	0.22	0.13	0.36	0.26	0.19	0.15	0.00									
NI	0.22	0.40	0.30	0.17	0.18	0.12	0.20	0.20	0.16	0.11	0.32	0.22	0.15	0.07	0.10	0.00								
TF	0.29	0.44	0.42	0.24	0.27	0.21	0.28	0.28	0.26	0.23	0.37	0.27	0.21	0.27	0.24	0.20	0.00							
GJ	0.52	0.70	0.65	0.46	0.49	0.44	0.51	0.50	0.49	0.46	0.58	0.50	0.46	0.50	0.48	0.44	0.48	0.00						
SG	0.54	0.74	0.68	0.49	0.52	0.47	0.55	0.53	0.53	0.49	0.62	0.53	0.49	0.53	0.50	0.47	0.51	0.74	0.00					
GO	0.42	0.64	0.58	0.39	0.41	0.34	0.42	0.41	0.39	0.38	0.51	0.44	0.39	0.40	0.40	0.35	0.42	0.65	0.68	0.00				
GR	0.34	0.55	0.51	0.31	0.34	0.26	0.28	0.27	0.27	0.29	0.43	0.35	0.29	0.32	0.31	0.26	0.34	0.57	0.58	0.45	0.00			
DH	0.37	0.52	0.49	0.32	0.36	0.29	0.36	0.36	0.34	0.30	0.43	0.35	0.29	0.36	0.32	0.29	0.33	0.55	0.60	0.50	0.42	0.00		
UJ	0.52	0.64	0.66	0.46	0.50	0.44	0.53	0.52	0.49	0.46	0.58	0.48	0.36	0.52	0.46	0.44	0.43	0.72	0.77	0.69	0.59	0.53	0.00	
YD	0.35	0.52	0.48	0.30	0.33	0.26	0.33	0.33	0.32	0.27	0.41	0.33	0.29	0.33	0.31	0.27	0.31	0.53	0.50	0.47	0.38	0.39	0.54	0.00

See **Table 1** for the population abbreviations. All values are statistically significant at P < 0.01.

**Table 3:** Summary of Bottleneck results for 24 *Sicyos angulatus* populations in South Korea based on both Wilcoxon and sign tests under the infinite allele model (IAM) and stepwise mutation model (SMM).

Population	*P* (sign test)		*P* (Wilcoxon test)		Mode shift
	IAM	SMM	IAM	SMM	
YF	0.000	0.000	0.000	0.000	Y
YT	0.000	0.000	0.996	1.000	N
GF	0.003	0.100	0.000	0.259	N
GS	0.000	0.000	0.000	0.000	Y
GT	0.000	0.000	0.000	0.000	Y
HF	0.000	0.000	0.000	0.000	Y
HS	0.000	0.000	0.000	0.000	Y
HT	0.000	0.000	0.000	0.000	Y
HO	0.000	0.000	0.000	0.000	Y
HI	0.000	0.000	0.000	0.000	Y
HX	0.000	0.000	0.000	0.000	Y
NF	0.000	0.000	0.000	0.000	Y
NS	0.000	0.000	0.000	0.000	Y
NT	0.000	0.000	0.000	0.000	Y
NO	0.000	0.000	0.000	0.000	Y
NI	0.000	0.000	0.000	0.000	Y
TF	0.000	0.000	0.000	0.000	Y
GJ	0.000	0.000	1.000	1.000	Y
SG	0.000	0.000	0.000	0.000	Y
GO	0.000	0.000	0.000	0.000	Y
GR	0.000	0.000	0.000	0.000	Y
DH	0.008	0.409	0.000	0.486	Y
UJ	0.000	0.004	0.000	0.001	Y
YD	0.276	0.300	0.035	0.771	Y

Results of the mode-shift test is also presented. A shifted mode is expected when population bottlenecks are present.

**Figure 3:**
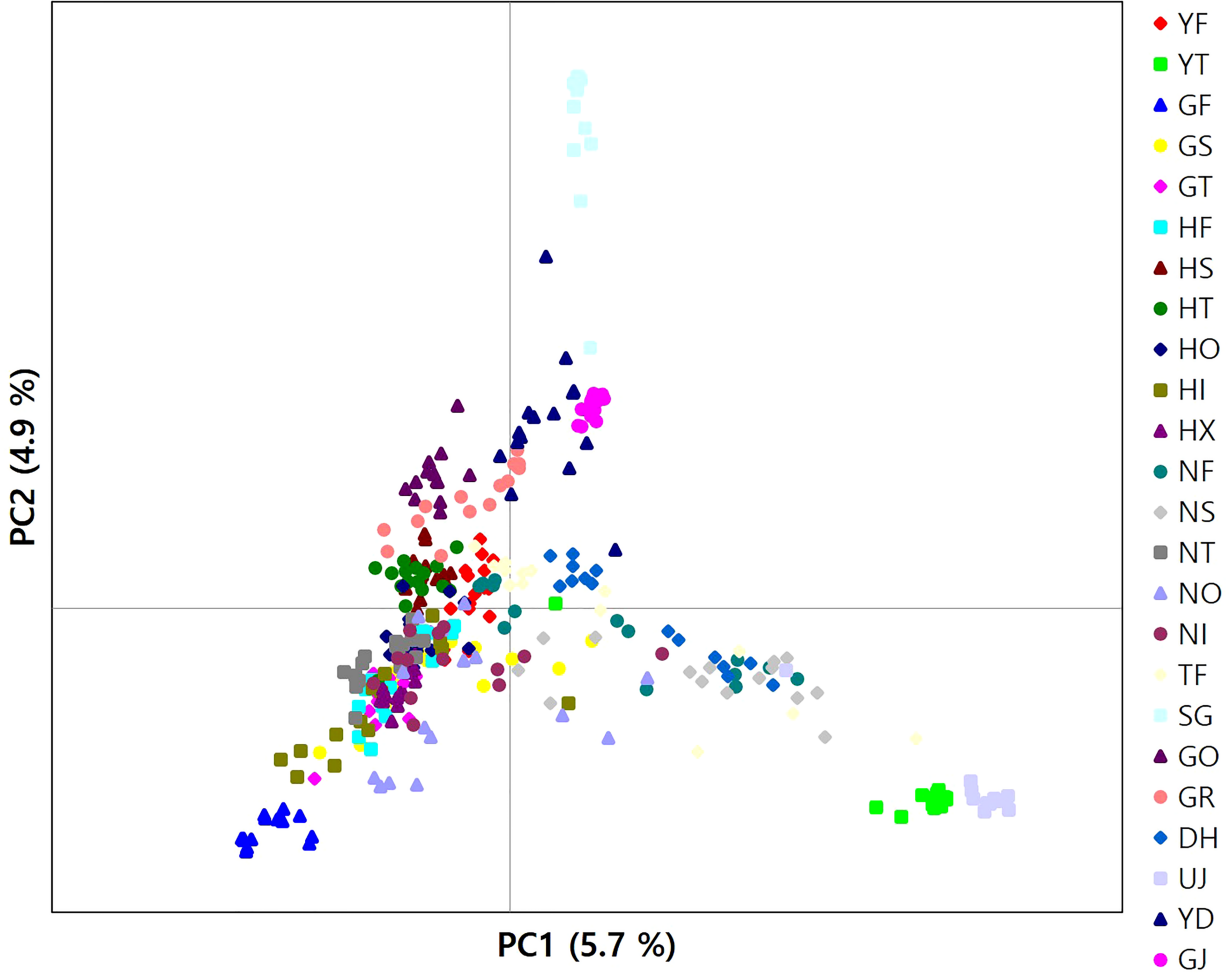
A plot of principal components analysis for 346 burcucumber genotypes. The first two variance components were plotted.

The results of clustering analyses differ between PCoA and fastSTRUCTURE (Figs. 1 and 3). In our PCoA analysis, the first three axes only accounted for ~15% variation (**Figure 3**). UJ and YT were clearly separated from the remaining populations along the first axis, yet the variation explained by PC1 was about 6% (plots associated with second and third provided in **Figure S1**). The second axis (PC2 = 4.9%) separates SG populations from the rest, whereas SG and GJ were clustered and departed from the rest of the populations along the third axis (PC3 = 4.3%; **Figure 3**). The optimal *K*, the number of clusters at HWE, inferred from FastSTRUCTURE ranged from 2 to 7 (*K* = 3 presented in **Figure 1**; plots for *K* = 2–7 provided in **Figure S2**). The overall pattern of clustering reflected the originated river basin, and it was consistent throughout the varying cluster numbers with slight differences as *K* increases (**Figure S2**). Here, we summarized the result of the clustering pattern for *K*3 since it offers a simple and spatially more intuitive visual presentation. Overall in *K*3, populations within the river basins shared similar assignment patterns forming a deme (**Figure 1**). The HG, GG, and ND river basins (see **Table 1** for the river basin acronyms) were primarily assigned to a cluster represented in red, but a small fraction of the genomic composition was also affiliated with the remaining two clusters (**Figure 1**). The two populations in the YS river basin showed a contrasting assignment pattern. YF shared a rather similar pattern with the populations within the HG, GG, and ND river basins, whereas YT was much alike with populations within the DR, the east coastal streams (**Figure 1**). Populations in DR streams exhibited a little more complex pattern than populations located in the rest river basins, which are composed of more admixed genotypes with all the three genetic clusters (**Figure 1**). In *pPost-hoc* AMOVA analyses associated with the FastSTRUCTURE clustering pattern, the average population differentiation across the river basins (the percentage variation explained = 4.5%; F*_CT_* = 0.05, *P* < 0.01) was much less than the population differentiation within each river basin (the percentage variation explained = 32%; F*_SC_* = 0.34, *P* < 0.01; **Table 4**).

**Table 4:** Results of AMOVA partitioning the genetic variation of *Sicyos angulatus* in Korea representing 16 populations within four river basins and eight populations from east coastal streams (see **Table 1** for the regions).

Source	Sum of squares	Variance components	Percentage of variation	Fixation index
Among groups (FCT)	35,886.396	20.859	4.467	0.045
Among population within groups (FSC)	86,044.127	151.422	32.428	0.339
Among individuals within populations (FIS)	139,311.945	137.973	29.547	0.468
Within individuals (FIT)	54,218.000	156.699	33.558	0.664

All variance components were statistically robust (P < 0.01).

Based on the LDA plot for the prior model checking, the location of the observed data was within the range of the simulated data, which suggests that ca. 3,000 simulated data sets per model were suitable for inferring the model choice (**Figure S3A**). Likewise, the out-of-bag error rates were stabilized with an increasing number of trees indicating that the number of trees produced for each model was sufficient (**Figure S3B**). The classification vote was the highest in scenario 7 (195) with a posterior probability of 0.67, which was followed by scenario 4 (127). The global and local errors were 0.093 and 0.156, respectively. The DIYABC-RF analysis strongly suggested that there were multiple introductions from two genetically divergent sources during the Korean cucumber invasion (see scenario 7 in **Figure 2**). The time of introduction (t1) was estimated approximately 70 years, whereas the divergence time in origin for the two genetically distinct sources dated back a little less than 300 years (**Table 5**).

**Table 5:** Summary results of DIYABC-rf parameter estimation under scenario 7.

Parameter	Mean	Median	90% CI	Global (prior) NMAE computed from		Local (posterior) NMAE computed from	
				Mean	Median	Mean	Median
ta	268.219	264.817	(134.332, 444.979)	0.293	0.298	0.381	0.367
t1	72.3884	74	(32.0, 100.0)	0.243	0.246	0.294	0.376
N1	500.175	446.326	(114.0, 957.589)	0.351	0.339	0.968	1.154
N2	638.931	703.811	(130.0, 984.0)	0.367	0.371	0.781	0.766
N3	344.284	247	(105.0, 807.0)	0.294	0.274	0.465	0.228
N4	3,697.32	3,275.23	(765.684, 9,332.04)	1.042	0.827	1.153	0.95
N5	5,114.84	5325	(438.404, 9,440.6)	2.599	2.229	2.884	1.339

See detailed scenario information in **Figure 2**.

With DEM and bioclimatic variables, we developed the best-fitting species distribution model and prediction using the maximum entropy method for the burcucumber (MaxEnt; AUC = 0.83 ± sd 0.012; **Figure S4**). In the model, the most contributed variable was B19 (precipitation of the coldest quarter; 33.7% of total contribution), followed by DEM (21.8%), B17 (precipitation of the driest quarter; 6.4%), and B13 (precipitation of the wettest month; 5.8%, **Table S1**). The contributions of other variables for the model were below 5%. The optimal habitats inferred from the SDM were distributed around big cities with a river, where high disturbances are expected (**Figure S5**). The resistance distance estimated varied greatly across population pairs (ranging from 7, TF/GJ to 105, YT/GO; **Table S2**). In our MLPE models, models with F*_ST_* outperform the linearized F*_ST_* whereas the three different distances computed showed a similar model fit (**Table S3**). The model fits of the linearized F*_ST_* dropped about 20% compared with the F*_ST_*, and the confidence intervals for the correlation coefficient values include zeros for all three distance models (**Table S3**). The best predictor of the genetic discontinuity was the log-transformed Euclidean distance (**Table 6**). Models associated with resistance distance showed low support (ΔcAIC ≥ 10; Burnham and Anderson, 2002).

**Table 6:** Summary of maximum likelihood population effect (MLPE) analysis.

Model	Euclidean	logEuclidean	Resistance	logLike	cAIC	ΔcAIC
1	NA	0.0450	NA	498.8611	-987.4999	0.0000
2	0.0109	NA	NA	491.9936	-973.7650	13.7350
3	NA	NA	0.0005	491.1729	-972.1236	15.3764

The models with three different predictor distances are presented. For each model, the fixed effect slope (β), log likelihood (logLike), and cAIC values are provided. Missing cells indicate that the distance element was not used in the model. logEuclidean refers to the log-transformed Euclidean distances between a pair of populations. NA, not available.

## Discussion

The major components of successful colonization consist of high propagule pressure, multiple introductions, release from natural enemies, and competitive life history traits (Sakai et al., 2001; Theoharides and Dukes, 2007; Hirsch et al., 2017). Despite the short colonization period (less than a half-century) and supposedly low propagule pressure during the initial introduction, burcucumber invasion in Korea has been a great success (National Institute of Environmental Research, 2005). However, given the lack of well-documented records, the invasion history of burcucumber has been largely unknown. Through molecular and landscape data, we partly reconstructed invasion paths of burcucumber in Korea. The ABC and Bayesian assignment results revealed that the burcucumber invasion has been tightly associated with multiple introductions from more than two genetically divergent sources from the origin. In contrast, the landscape genetics results indicated that landscape factors did not primarily contribute to the burcucumber invasion in Korea.

Most populations investigated in the study harbored rather low genetic diversity (**Table 1**). The genetic diversity observed in our study was a little lower than or comparable with the ones measured from other invasive plants. For instance, the expected heterozygosity observed (mean *He* = 0.17) in the study was lower than the long-lived invasive shrubs’ [*Tamarix ramosissima*, mean *He* based on 1,996 SNPs = 0.30 (Lee et al., 2018); *Frangula alnus*, mean *He* based on 133 SNPs = 0.29 (de Kort et al., 2016)], an invasive perennial herb’s [*Reynoutria japonica*, mean *He* based on 12,912 SNPs = 0.30 (VanWallendael et al., 2021)], and an annual exotic’s [*Mimulus guttatus*, mean *He* based on 69 SNPs = 0.32 (Pantoja et al., 2017)]. The reduced genetic variability was a little surprising as the mating system of burcucumber is mostly outcrossing (Arifin and Okamoto, 2020). Outcrossing plants, in general, are genetically more diverse than self-compatible plants (Hamrick and Godt, 1996). Due to lack of native samples, the direct comparison between native and the introduced populations of burcucumber could not be carried out in this study. Accordingly, extreme care must be taken when interpreting the lowered genetic diversity. Nevertheless, considering the recent population bottlenecks observed from most populations examined in South Korea (**Table 4**), the low genetic diversity likely resulted from the founder effect that is commonly expected for a colonizing species.

The inflated genetic divergences among the regional populations [*S. angulatus* mean F*_ST_* = 0.37] might also be driven by the drastic decline in population sizes. F*_ST_*, the among-population differentiation measure used in the study, is inversely related to the effective population size as suggested by Wright (1943); Wright (1950); F*_ST_* ≈ 1/4*N_e_* m +1), which was empirically verified by a number of studies, e.g., Jordan and Snell (2008) and Whiteley et al. (2010). The small population size likely resulted from the founder effect might also be the cause of the increased F*_ST_* in our study. Alternatively, the mating system of burcucumber (mostly outcrossing by small insects, Arifin and Okamoto, 2020) might partly contribute to the high F*_ST_* values. Life history traits largely impact the genetic diversity pattern in both within- and among-population levels (Hamrick and Godt, 1996). A wind-pollinated long-lived tree, in general, exhibits high genetic diversity with reduced population differentiation, whereas a herbaceous plant with insect pollination often harbors relatively small genetic variation and high population divergence (Hamrick et al., 1981; Olson et al., 2016). Particularly, the herbs pollinated by small insects likely show greater among-population divergence, which may also be the causal mechanism of the increased population divergence observed in our study (Gamba and Muchhala, 2020).

The spatial clustering pattern suggests multiple introductions of divergent lineages during burcucumber invasion in Korea. In *K* = 3, four populations (GF, GJ, YT, and NT) were assigned nearly to a single cluster, suggesting that those populations might be the sources of an early establishment (**Figure 1B**). Except for YS, populations within the same river basin shared a similar clustering pattern (**Figure 1**). Contrary to the assignment pattern of the populations within a river basin, we found an abrupt shift in population allele frequencies between YF and YT along the YS river basin, which is repeated between TF and GJ, neighboring populations in DR streams (**Figure 1**). The burcucumber fruits carrying water-impermeable seeds (Walker, 1973) can travel by floating on water stream, thus contributing to gene flow among populations within a river basin. Considering the geographic proximity, the abrupt change of allele frequency observed between the two neighboring populations along the YS river basin and DR streams was a rather surprising result. The steep allele frequency change likely resulted from human interventions such as repeated independent introductions from multiple sources that are genetically diverse. The pattern of the multiple introductions coincides with the historical records, although those records are incomplete and insufficient to reconstruct the whole burcucumber invasion in Korea. Notably, in the occurrence records, we found that after just 1 year since the first report in 1989, the burcucumber was recorded in several locations throughout South Korea (National Institute of Environmental Research, 2005). The multiple occurrences at such large spatial scale within a year may only be possible by multiple introductions. Alternatively, varying selection regimes responding to sudden environmental changes between the neighboring areas might result in similar genetic discontinuities. However, the effects of selection tend to affect relatively small regions of the genome (e.g., tb1 in maize; Kaplan et al., 1989; Andolfatto, 2001; Charlesworth et al., 2003; Baucom, 2016). Therefore, genetic discontinuity assessed from genome-wide molecular markers in our study is more likely derived from demographic factors such as multiple introductions (Black et al., 2001).

Consistent with the spatial clustering pattern (**Figure 1**) that we observed, the best model inferred from DIYABC results strongly supported the multiple introductions. The scenario of choice demonstrated that there were at least two genetically divergent sources introduced from the origin (**Figure 2** scenario 7). According to the selected scenario, the two distinct sources diverged into the clustering groups represented by red and green after the initial colonization (see **Figure 1** for the cluster colors). Subsequently, the dominant red group has likely spread into the middle west (GS and GF) and diverged into the blue group along the east coastal area while passing through the middle area (NS, NF; Figs. 1 and 2 scenario 7). Although the selected scenario shed some light on the path of the burcucumber invasion in Korea such as a strong association with multiple introductions, some of the detailed assessments should be interpreted with caution. For example, the number of genetically distinct sources may not be precise as we constructed the scenarios with summarized clustering groups based on K = 3. In fact, scenario 4 (**Figure 2** scenario 4) which assumes the three independent introductions and subsequent divergences to the clustering groups represented by red, blue, and green showed the second highest classification votes (votes for scenario 7 = 195; votes for scenario 4 = 127). Considering the lack of genotype data from the origin, the detailed demographic history inferred in our study was not without caveats. However, by employing the ABC approach, we were able to determine the tight association of the multiple introductions with the burcucumber invasion in Korea.

The result of our landscape genetics models was not consistent with what was expected from the previous studies. Because circuit theory-based IBR accounts for spatial heterogeneity, IBR, in general, outperforms the conventional IBD (McRae, 2006; McRae and Beier, 2007; Emel et al., 2021). However, in our landscape genetics model, circuit resistance was not the best predictor of genetic discontinuity, suggesting a general lack of environmental effects on population divergence. The resistance distance was computed based on the SDM; thus, the resistance is primarily attributed to the temperature-related variables and DEM, which is also correlated with the temperature. Given the temperature-dependent germination and growth habits of burcucumber (Walker, 1973; Smeda and Weller, 2001; Onen et al., 2018), it was unexpected that the resistance distance was not the key predictor of the population divergences. The repeated introductions from genetically divergent sources might have driven the weak environmental effects. Smith et al. (2020) provided a compelling example of the reduced effect of environmental gradient on genetic structure in the non-native range. Consistent with our results, the most significant predictor of genetic discontinuity for a widespread weed, *Plantago lanceolata*, was the geographic distance in the colonizing area, whereas native ones were influenced more by environmental factors (Smith et al., 2020). Coupled with the population bottlenecks found in our study, multiple introductions and the genetic admixture likely lowered the environmental effects on the spatial structure among populations. With the aid of demographic events, burcucumber populations might have rapidly expanded their range without a need for adaptation to the local environments. Some might argue that our landscape genetics approach does not consider the underlying assumption, the gene flow-drift equilibrium among populations (Marko and Hart, 2011; Epps and Keyghobadi, 2015). However, the equilibrium is an unlikely scenario for an invasive plant with the rapid and recent history of colonization such as the burcucumber that invaded in Korea.

## Conclusion

Burcucumber invasion in Korea has been tightly linked to a suite of factors including the continued propagule pressure from multiple and deliberate and/or accidental introductions from unknown seed sources, unplanned human aids in dispersal, founder effects, river network effects, and maybe countless unaccounted factors. As shown in a number of empirical studies (Hirsch et al., 2017; van Boheemen et al., 2017; Bertelsmeier and Keller, 2018), multiple introductions vectored by anthropogenic causes enhance the propagule pressure and strongly influence the adaptive potential for a widespread invasive species (Simberloff, 2009; Dlugosch et al., 2015; Bertelsmeier and Keller, 2018; Blackburn et al., 2020). Not all invasive species, therefore, suffer from environmental constraints in the introduced range; instead, a merger of divergent genetic lineages from multiple populations can ameliorate the adaptive challenges. Our work thus addresses the importance of preventing secondary introductions, particularly for aggressive weedy plants like burcucumber. A flow of continued propagule pressure and a chance of admixture through the human-mediated secondary introductions may increase the chance of further range expansion in Korea. It is highly recommended to build a periodic and well-planned monitoring system for the new introductions particularly to the ports along the East and West Seas. Preventing further introductions of burcucumber must be the number one priority in management strategy in Korea. We also hope that our study motivates to further investigate the genomic and population-level diversity pattern of the native region and fully reconstruct the invasion paths.

## Data availability statement

The datasets presented in this study can be found in online repositories. The names of the repository/repositories and accession number(s) can be found below: https://www.ncbi.nlm.nih.gov/genbank/, PRJNA822832.

## Author contributions

S-RL and DCS conceived ideas and designed the study. DCS prepared research grant. Field sampling was planned and performed by S-RL. S-RL designed the laboratory work and performed the genetic and statistical analyses. S-RL wrote the manuscript. DCS edited the manuscript. All authors contributed to the article and approved the submitted version.

## Funding

This work was supported by a research project of the Korea National Arboretum (KNA1-2-39, 21-2).

## Acknowledgments

We are grateful to a postdoctoral fellow, Tae-Young Choi, for the laboratory and analytical assistance. We also thank the graduate assistant, Eun-Su Kang, with the undergraduate assistants, Tea-Young Eom, Hyeon-Su Kim, and Geon-Ho Kim, for their help with field sampling and laboratory work.

## Conflict of interest

The authors declare that the research was conducted in the absence of any commercial or financial relationships that could be construed as a potential conflict of interest.

## Publisher’s note

All claims expressed in this article are solely those of the authors and do not necessarily represent those of their affiliated organizations, or those of the publisher, the editors and the reviewers. Any product that may be evaluated in this article, or claim that may be made by its manufacturer, is not guaranteed or endorsed by the publisher.
