# Supplementary material for: Genetic diversity pattern reveals the primary determinant of burcucumber (Sicyos angulatus L.) invasion in Korea
Source: Front Plant Sci. 2022 Nov 15;13:997521. doi: 10.3389/fpls.2022.997521 (PMC9706109; doi:10.3389/fpls.2022.997521)
Supplement: Supplementary file 1 [file DataSheet_1.docx]

Supplementary Information

Genetic diversity pattern reveals the primary determinant of burcucumber (*Sicyos angulatus* L.) invasion

Soo-Rang Lee^1*^ (SRL) and Dong-Chan Son (DCS) ^2^

^------------------------------------------------------------------------------------------------^

^1^Department of Biology Education, College of Education, Chosun University, Gwangju 61452, South Korea

^2^Division of Forest Biodiversity and Herbarium, Korea National Arboretum, Pocheon 11186, Republic of Korea

Figure S1. Plots of principal components analysis for 346 burcucumber genotypes. The first three variance components were plotted.


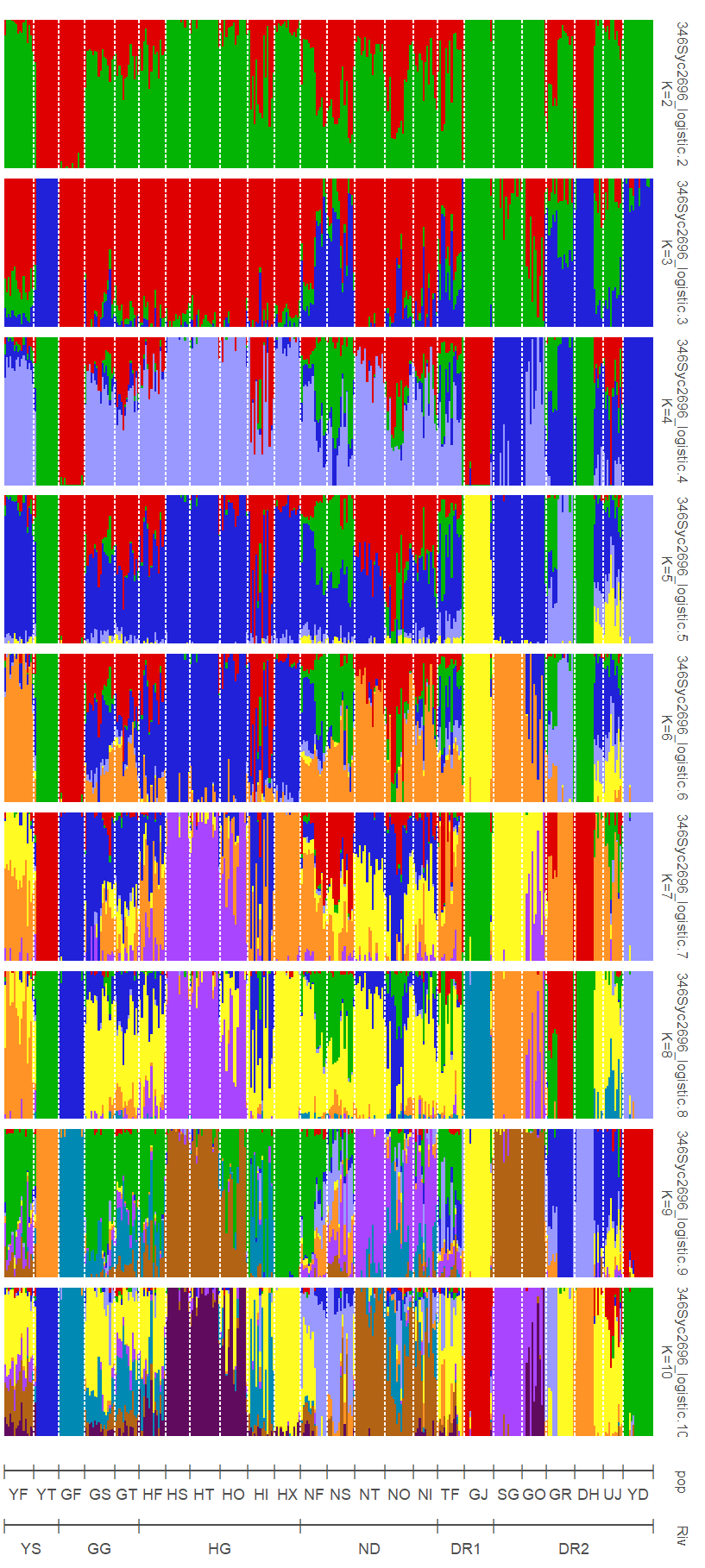

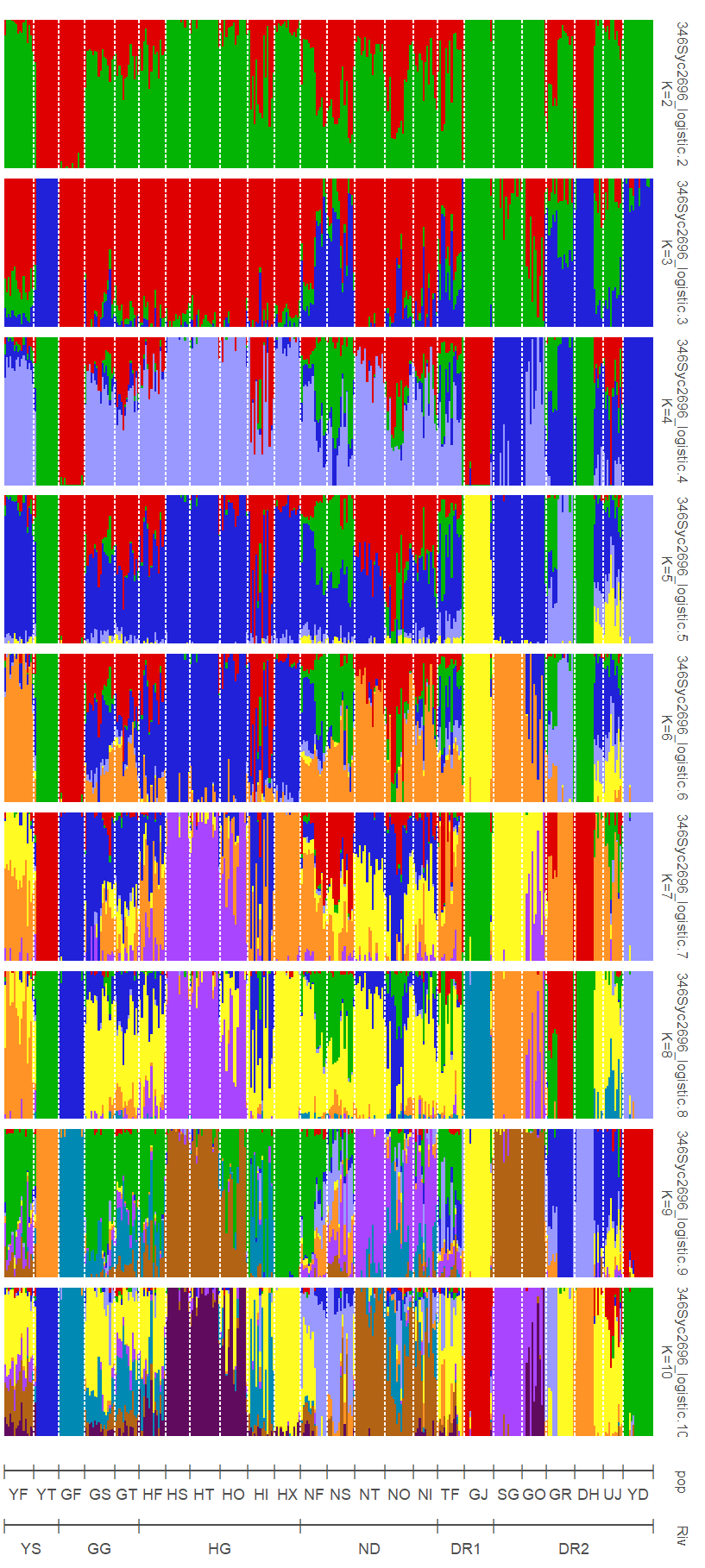


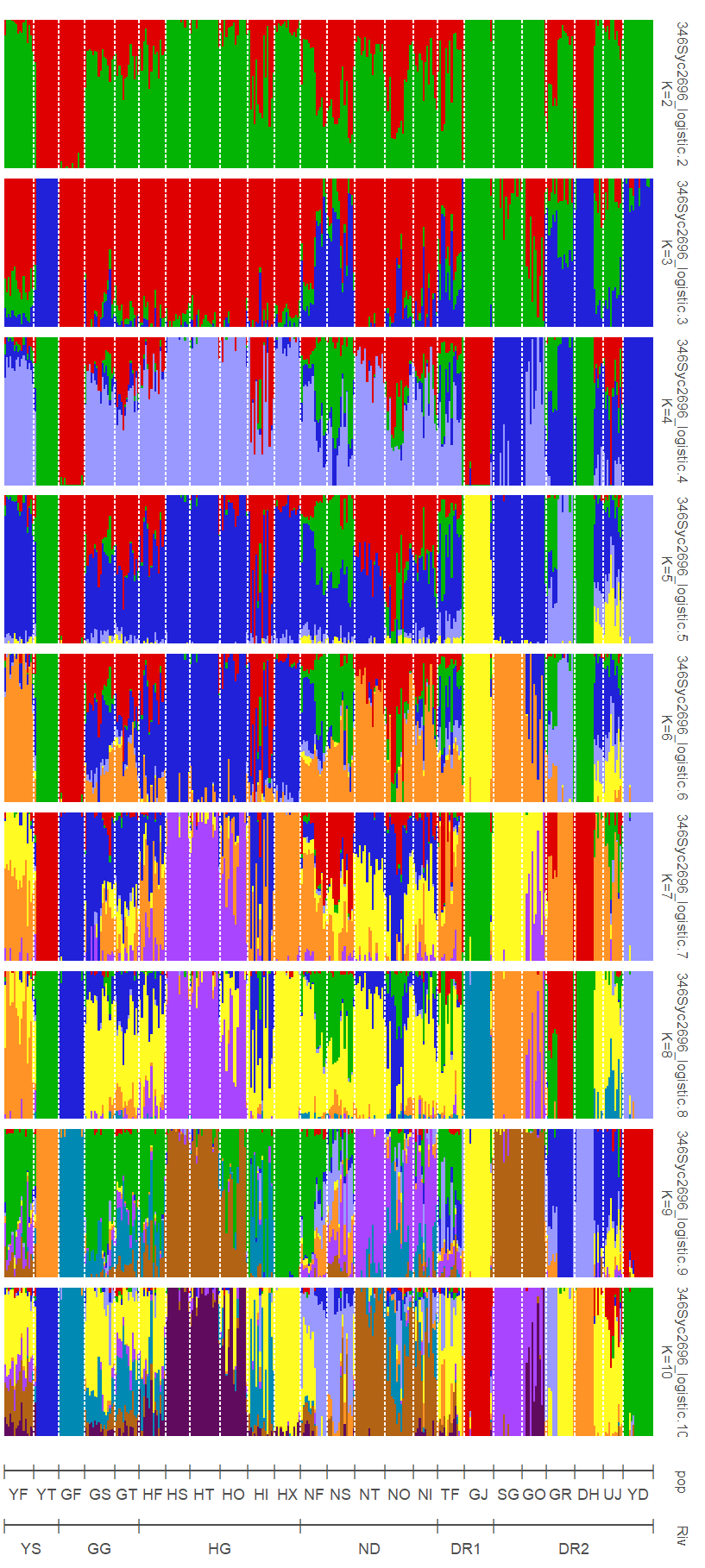


Figure S2. Bar charts of the clustering assignment for the *K* = 2 - *K* = 10 from fastSTRUCTURE based on 2696 SNPs sampled across 346 burcucumber genotypes. Populations are separated by dotted vertical lines.

The upper bottom scale bars indicate the populations assigned and the lower bottom scale bars refer to the river basins each population belongs to. Each color represents an assignment of loci into each clustering group for the *K* =2 - 10. See Table 1 for population abbreviations.

a)


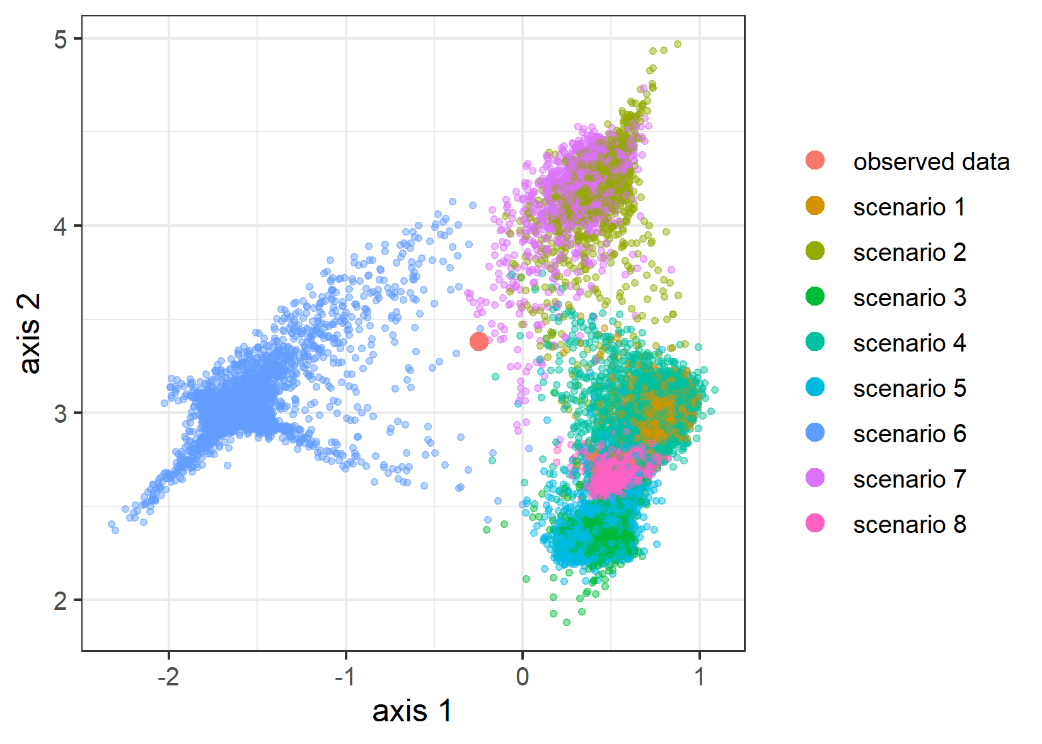


b)


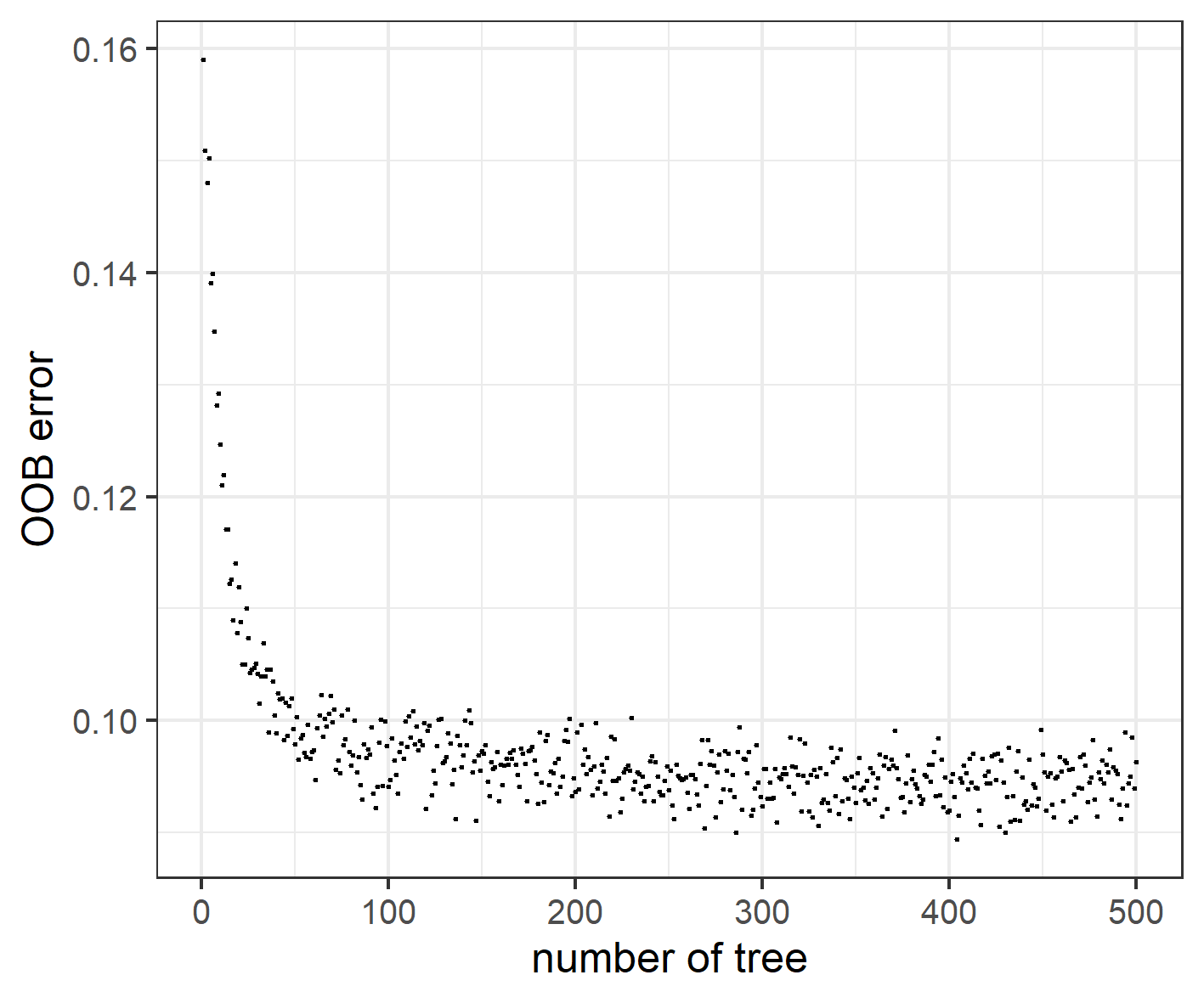


Figure S3. DIYABC-RF model checking. a) A plot for the first two axes of the linear discriminant (LDA) analysis from the training datasets. The observed data (orange circle) was within the simulated prior distribution of parameters under the eight scenarios. b) The out of bag error rate over the number of trees in the forest indicating that 3,000 simulated data we used was a sufficient number of trees to sample for the Random Forest Analysis.
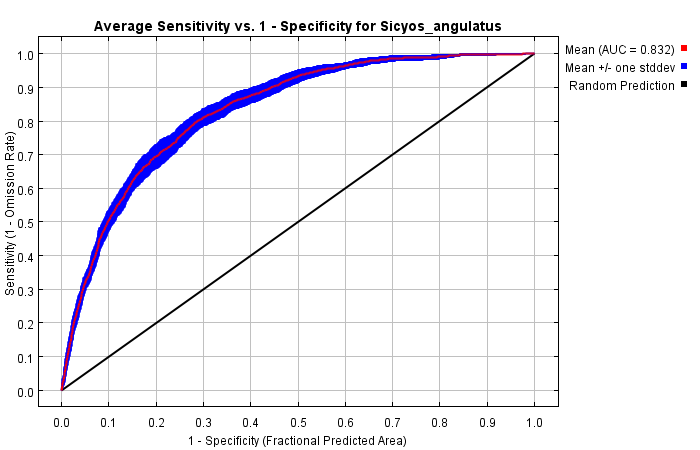


Figure S4. The receiver operating characteristic (ROC) curve for habitat suitability model of Sicyos angulatus in South Korea. AUC (Area under the ROC curve) value above 0.5 supports the model.


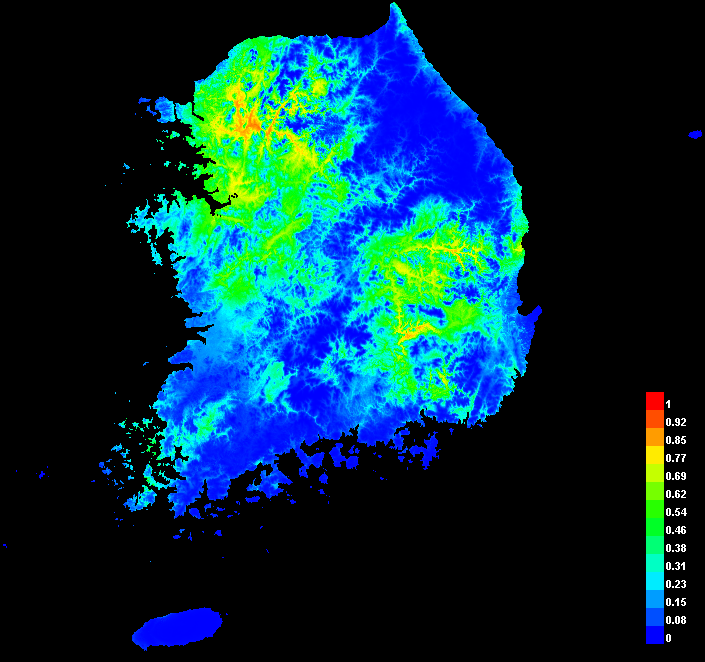


Figure S5. The habitat suitability model of *Sicyos angulatus* in South Korea. Red color (close to 1) means more suitable habitats, while blue (close to 0) represents less suitable habitats.

Table S1. Variable contribution for the habitat suitability model of *Sicyos angulatus* in South Korea.

| **Variable** | **Percent contribution** |
| --- | --- |
| Precipitation of Coldest Quarter (BIO19) | 33.7 |
| Elevation | 21.8 |
| Precipitation of Driest Quarter (BIO17) | 6.4 |
| Precipitation of Wettest Month (BIO13) | 5.8 |
| Precipitation Seasonality (BIO15) | 4.2 |
| Temperature Seasonality (BIO4) | 3.1 |
| Annual Mean Temperature (BIO1) | 2.9 |
| Temperature Annual Range (BIO7) | 2.8 |
| Mean Diurnal Range (BIO2) | 2.7 |
| Mean Temperature of Wettest Quarter (BIO8) | 2.6 |
| Mean Temperature of Warmest Quarter (BIO10) | 2.5 |
| Min Temperature of Coldest Month (BIO6) | 2.4 |
| Precipitation of Warmest Quarter (BIO18) | 1.6 |
| Isothermality (BIO3) | 1.6 |
| Max Temperature of Warmest Month (BIO5) | 1.6 |
| Annual Precipitation (BIO12) | 1.6 |
| Precipitation of Driest Month (BIO14) | 1.3 |
| Mean Temperature of Driest Quarter (BIO9) | 0.9 |
| Precipitation of Wettest Quarter (BIO16) | 0.4 |
| Mean Temperature of Coldest Quarter (BIO11) | 0 |

Table S2. The pairwise resistance distances computed from Circuitscape based on the reversed habitat suitability estimated by Species Distribution Model.

|  | YF | YT | GF | GS | GT | HF | HS | HT | HO | HI | HX | NF | NS | NT | NO | NI | TF | GJ | SG | GO | GR | DH | UJ | YD |
| --- | --- | --- | --- | --- | --- | --- | --- | --- | --- | --- | --- | --- | --- | --- | --- | --- | --- | --- | --- | --- | --- | --- | --- | --- |
| YF | 0.00 |  |  |  |  |  |  |  |  |  |  |  |  |  |  |  |  |  |  |  |  |  |  |  |
| YT | 7.68 | 0.00 |  |  |  |  |  |  |  |  |  |  |  |  |  |  |  |  |  |  |  |  |  |  |
| GF | 40.34 | 47.17 | 0.00 |  |  |  |  |  |  |  |  |  |  |  |  |  |  |  |  |  |  |  |  |  |
| GS | 39.97 | 46.61 | 13.38 | 0.00 |  |  |  |  |  |  |  |  |  |  |  |  |  |  |  |  |  |  |  |  |
| GT | 25.47 | 30.55 | 27.59 | 17.67 | 0.00 |  |  |  |  |  |  |  |  |  |  |  |  |  |  |  |  |  |  |  |
| HF | 69.40 | 76.02 | 44.96 | 35.69 | 45.67 | 0.00 |  |  |  |  |  |  |  |  |  |  |  |  |  |  |  |  |  |  |
| HS | 90.33 | 96.97 | 50.52 | 50.36 | 67.47 | 27.05 | 0.00 |  |  |  |  |  |  |  |  |  |  |  |  |  |  |  |  |  |
| HT | 80.89 | 80.53 | 43.23 | 40.92 | 58.03 | 20.43 | 9.44 | 0.00 |  |  |  |  |  |  |  |  |  |  |  |  |  |  |  |  |
| HO | 70.94 | 77.58 | 38.38 | 30.97 | 48.08 | 12.08 | 19.39 | 9.95 | 0.00 |  |  |  |  |  |  |  |  |  |  |  |  |  |  |  |
| HI | 68.45 | 75.28 | 28.11 | 28.76 | 45.91 | 24.20 | 22.42 | 15.12 | 12.12 | 0.00 |  |  |  |  |  |  |  |  |  |  |  |  |  |  |
| HX | 61.34 | 68.17 | 21.00 | 25.33 | 43.00 | 34.74 | 32.46 | 26.18 | 22.66 | 11.07 | 0.00 |  |  |  |  |  |  |  |  |  |  |  |  |  |
| NF | 70.15 | 77.83 | 34.71 | 44.28 | 61.95 | 59.12 | 49.03 | 46.57 | 47.04 | 35.22 | 24.38 | 0.00 |  |  |  |  |  |  |  |  |  |  |  |  |
| NS | 51.05 | 58.73 | 17.61 | 28.53 | 45.20 | 51.05 | 49.74 | 43.46 | 39.68 | 28.35 | 17.29 | 19.19 | 0.00 |  |  |  |  |  |  |  |  |  |  |  |
| NT | 50.06 | 57.74 | 27.01 | 39.99 | 47.89 | 67.18 | 68.41 | 63.31 | 59.28 | 48.19 | 37.12 | 29.47 | 19.84 | 0.00 |  |  |  |  |  |  |  |  |  |  |
| NO | 45.43 | 53.11 | 32.64 | 45.62 | 53.52 | 77.60 | 80.34 | 74.62 | 70.48 | 59.50 | 48.44 | 41.24 | 31.76 | 11.92 | 0.00 |  |  |  |  |  |  |  |  |  |
| NI | 59.37 | 66.43 | 47.39 | 60.37 | 68.27 | 89.37 | 90.60 | 85.49 | 81.46 | 70.37 | 59.31 | 43.83 | 42.03 | 22.19 | 14.75 | 0.00 |  |  |  |  |  |  |  |  |
| TF | 68.59 | 76.27 | 50.48 | 63.46 | 71.36 | 89.50 | 83.56 | 80.42 | 78.00 | 66.41 | 55.35 | 36.21 | 38.46 | 23.47 | 23.16 | 12.44 | 0.00 |  |  |  |  |  |  |  |
| GJ | 69.57 | 77.22 | 47.79 | 60.77 | 68.67 | 83.91 | 76.53 | 73.39 | 71.83 | 60.01 | 49.17 | 29.18 | 34.41 | 20.78 | 24.14 | 18.55 | **7.03** | 0.00 |  |  |  |  |  |  |
| SG | 70.76 | 77.59 | 30.42 | 39.63 | 57.30 | 43.24 | 30.49 | 28.64 | 31.16 | 19.04 | 14.30 | 18.54 | 20.38 | 37.92 | 49.85 | 60.11 | 53.35 | 46.32 | 0.00 |  |  |  |  |  |
| GO | 97.57 | 105.04 | 57.87 | 63.89 | 81.33 | 49.11 | 22.05 | 30.85 | 40.07 | 37.34 | 39.46 | 40.90 | 46.58 | 64.12 | 76.04 | 83.28 | 73.17 | 66.14 | 29.42 | 0.00 |  |  |  |  |
| GR | 87.41 | 94.88 | 47.71 | 57.28 | 74.95 | 53.83 | 32.75 | 36.51 | 42.08 | 36.24 | 35.01 | 27.05 | 36.41 | 53.96 | 65.88 | 69.42 | 59.26 | 52.23 | 20.93 | 21.26 | 0.00 |  |  |  |
| DH | 80.11 | 87.58 | 40.40 | 49.98 | 67.65 | 59.72 | 39.11 | 42.87 | 47.97 | 39.06 | 30.08 | 19.18 | 29.11 | 46.65 | 58.58 | 61.04 | 49.85 | 42.82 | 21.86 | 28.51 | 12.69 | 0.00 |  |  |
| UJ | 82.37 | 89.84 | 42.67 | 52.24 | 69.92 | 67.08 | 51.30 | 54.26 | 55.00 | 43.19 | 32.35 | 17.25 | 31.38 | 42.56 | 53.71 | 51.54 | 40.36 | 33.33 | 26.11 | 40.78 | 26.80 | 14.88 | 0.00 |  |
| YD | 82.74 | 90.42 | 47.76 | 57.33 | 75.00 | 72.17 | 58.19 | 59.35 | 60.09 | 48.27 | 37.43 | 16.59 | 32.81 | 32.67 | 42.29 | 39.34 | 27.50 | 20.79 | 31.19 | 47.80 | 33.88 | 24.48 | 13.50 | 0.00 |

Table S3. Results of maximum likelihood population effects (MLPE) analysis. The models with three different predictor distances and population divergence parameters (F*_ST_* and linearized F*_ST_*) are presented. For each model, the fixed effects slope (β), the confidence intervals, the significance of the slope and the marginal r^2^ values are provided.

| Spatial distance metric | Genetic distance metric | | | | | |
| --- | --- | --- | --- | --- | --- | --- |
|  | F*_ST_* | | | Linearized F*_ST_* | | |
|  | r | p | r2 | r | p | r2 |
| Euclidean distance | 0.0109 [0.0046, 0.0172] | 0.001 | 0.921 | 0.0300 [-0.0104, 0.0703] | 0.000 | 0.773 |
| Ln(Euclidean distance) | 0.0449 [0.0269, 0.0629] | 0.000 | 0.918 | 0.0827 [-0.0358, 0.2005] | 0.170 | 0.773 |
| Resistance distance | 0.0005 [0.0002, 0.0007] | 0.000 | 0.922 | 0.0011 [-0.0004, 0.0026] | 0.158 | 0.773 |
